# A randomized controlled equivalence trial of pulmonary surfactant administration via laryngeal mask airway versus endotracheal intubation

**DOI:** 10.1186/s13063-025-09310-x

**Published:** 2025-12-02

**Authors:** Aoyu Wang, Chao Chen, Yi Huang, Wenli Liu, Ke Tian, Rong Zhou, Zhu Yuan, Jun Zhu, Yong Hu, Haiting Liu, Surong Duan, Hua Wang

**Affiliations:** 1 Department of Pediatrics, West China Second University Hospital, Sichuan University, No. 20, Section Three, South Renmin Road, Chengdu, China; 2 Key Laboratory of Birth Defects and Related Diseases of Women and Children of the Ministry of Education, Sichuan University, Chengdu, China; 3 Department of Clinical Medicine, BinZhou Medical College, Yantai, China

**Keywords:** Infant, Respiratory distress syndrome, Newborn, Pulmonary surfactants, Laryngeal masks, Intubation, Intratracheal

## Abstract

**Background:**

Neonatal respiratory distress syndrome (NRDS) is a life-threatening condition marked by progressive respiratory failure due to pulmonary surfactant (PS) deficiency, typically presenting within hours of birth. Exogenous pulmonary surfactant replacement therapy has significantly reduced mortality associated with NRDS and improved clinical outcomes. The current standard of care involves the intubation-surfactant-extubation (INSURE) technique. However, emerging evidence suggests that the laryngeal mask airway (LMA) may serve as a viable noninvasive alternative for neonatal resuscitation and therapeutic administration. This randomized controlled equivalence trial aims to compare the clinical efficacy and safety profiles of LMA and INSURE in delivering PS for NRDS management.

**Methods:**

Some of the preterm infants diagnosed with NRDS and requiring the first treatment with PS were included in this study. The included subjects were randomly assigned to the LMA group (experimental group) and the INSURE group (control group) by means of envelopes, and were given PS treatment by LMA or INSURE, respectively, and the SF index (SpO_2_/FiO_2_) before and after the use of PS; the placement time of artificial airway, the episodes of hypoxemia, bradycardia and tachycardia during artificial airway placement; the amount of PS residue in the stomach after the use of PS; the incidence of the second use of PS, and the incidence of pneumothorax were also monitored in order to compare the effect and prognosis of the two methods of treating NRDS.

**Discussion:**

The use of PS with INSURE is currently the most prominent and classic method. Although some recent studies suggest that non-invasive use of PS through a laryngeal mask is also effective, relevant research remains limited. Through this trial, we aim to gather more data regarding the use of PS with a laryngeal mask and further validate its effectiveness.

**Trial registration:**

Name of the registry: A randomized controlled trial of pulmonary surfactant administration via laryngeal mask airway versus endotracheal intubation.

Trial identifying number: ChiCTR2500096315.

Date of registration in primary registry: 2025-01-21 00:00:00.

PID: 254631.

Country of recruitment: China.

URL of trial registry record: https://www.chictr.org.cn/showproj.html?proj=254631.

## Administrative information

**Table:** 

Title {1}	A randomized controlled equivalence trial of pulmonary surfactant administration via laryngeal mask airway versus endotracheal intubation
Trial registration {2a and 2b}	Name of the registry: A randomized controlled trial of pulmonary surfactant administration via laryngeal mask airway versus endotracheal intubation Trial identifying number: ChiCTR2500096315 Date of registration in primary registry: 2025-01−21 00:00:00 PID: 254631 Country of recruitment: China URL of trial registry record: https://www.chictr.org.cn/showproj.html?proj=254631
Protocol version {3}	Version 2, Aug 11, 2023
Funding {4}	This study was funded by Sichuan Provincial Science and Technology Department Central Guidance Local Projects (2023ZYD0122) and National Natural Science Foundation of China (82241036). The funding organizations had no role in the study design, data collection, analysis, interpretation, or the publication of the manuscript
Author details {5a}	Aoyu Wang, West China Second University Hospital, Sichuan University, Chengdu, China. 550118451@qq.com Chao Chen, West China Second University Hospital, Sichuan University, Chengdu, China. chaochenrt@163.com Yi Huang, West China Second University Hospital, Sichuan University, Chengdu, China. huangxiaoyixx@outlook.com Wenli Liu, West China Second University Hospital, Sichuan University, Chengdu, China. 1179016063@qq.com Ke Tian, West China Second University Hospital, Sichuan University, Chengdu, China. 740690908@qq.com Rong Zhou, West China Second University Hospital, Sichuan University, Chengdu, China. 1490455039@qq.com Zhu Yuan, West China Second University Hospital, Sichuan University, Chengdu, China. yuanzhu716@163.com Jun Zhu, West China Second University Hospital, Sichuan University, Chengdu, China. 977343663@qq.com Yong Hu, West China Second University Hospital, Sichuan University, Chengdu, China. huyong1003@163.com Haiting Liu, West China Second University Hospital, Sichuan University, Chengdu, China. liuhaiting9@163.com Surong Duan, Department of Clinical Medicine, BinZhou Medical College, Yantai, China. 2687709707@qq.com Dr. Hua Wang, West China Second University Hospital, Sichuan University, Chengdu, China; Key Laboratory of Birth Defects and Related Diseases of Women and Children of the Ministry of Education, Sichuan University, Chengdu, China. wanghua@scu.edu.cn
Name and contact information for the trial sponsor {5b}	Dr. Hua Wang Tel: 86 13219080897 Email: wanghua@scu.edu.cn
Role of sponsor {5c}	The funding source had no role in the design of this study and will not be involved in its execution, data analysis, interpretation, or the decision to submit the results.

## Introduction

### Background {6a} {6b}

Pulmonary surfactant (PS) therapy, a groundbreaking advancement in neonatology, was pioneered by Fujiwara et al. in 1980 [1]. Over the past four decades, extensive clinical research has consistently validated the therapeutic efficacy of PS administration, demonstrating its remarkable ability to reduce neonatal mortality rates and enhance clinical outcomes in infants with respiratory distress syndrome (RDS) [2, 3]. In contemporary clinical practice, the intubation-surfactant-extubation (INSURE) technique remains the gold standard for PS administration. However, the field is currently undergoing significant evolution, with emerging noninvasive administration methods gaining increasing attention. These innovative approaches, including thin catheter delivery, laryngeal mask airway (LMA) application, and nebulization techniques, are either being actively implemented in clinical settings or undergoing rigorous investigation to optimize therapeutic delivery while minimizing invasive procedures [4–6].

The LMA has emerged as a promising noninvasive alternative for PS administration, with multiple clinical studies demonstrating its feasibility and therapeutic benefits. Research findings consistently indicate that LMA-assisted PS delivery significantly reduces supplemental oxygen requirements and decreases the need for mechanical ventilation in infants, offering a less invasive option compared to traditional methods [7–9]. These positive outcomes highlight the potential of LMA as a viable approach in neonatal respiratory care. However, despite these encouraging results, the current body of evidence remains limited, particularly in direct comparative studies evaluating the efficacy of LMA against the well-established INSURE procedure. This knowledge gap underscores the need for further rigorous research to establish the optimal application of LMA in PS administration protocols.

### Objectives {7}

In this study, we aimed to evaluate compare with INSURE, whether the administration of surfactant via LMA can be used as an effective alternative for the treatment of NRDS in preterm infants.

### Trial design {8}

This trial is a single-center randomized controlled trial. The included samples will be randomized into the experimental group (LMA group) and the control group (INSURE group) by methods of envelopes. The study is an equivalence trial designed to demonstrate that LMA and INSURE are equally effective in the treatment of NRDS using PS.

## Methods: participants, interventions, and outcomes

### Study setting {9}

This study will be conducted in the neonatal unit of West China Second Hospital, a teaching hospital in Chengdu, China.

### Eligibility criteria {10}

The inclusion and exclusion criteria of this study were primarily developed with reference to existing clinical studies on the use of LMA in infants with NRDS [7, 8, 10–15]. The specific criteria are as follows:

#### Inclusion criteria

1. Gestational age between 30 0/7 and 36 6/7 weeks, and/or birth weight ≥ 1500 g.
2. Age within 48 hours after birth.
3. Use of a noninvasive ventilator for more than 30 minutes.
4. Nasal continuous positive airway pressure (nCPAP), bi-level positive airway pressure (BiPAP), or non-invasive positive pressure ventilation (NIPPV) support ≥ 6 cmH_2_O, or high-flow nasal cannula (HFNC) support ≥ 6 L/min.
5. Need for fraction of inspired oxygen (FiO_2_) ≥ 0.30 to maintain pulse oximetry oxygen saturation (SpO_2_) levels between 90 and 94%.
6. Radiological evidence of RDS.

#### Exclusion criteria

1. History of prior invasive mechanical ventilation.
2. Previous PS therapy.
3. Congenital anomalies.
4. Pneumothorax.
5. Requirement for direct invasive ventilation.
6. Transfer from external hospitals.
7. Need for continued endotracheal intubation after the INSURE procedure.

### Who will take informed consent? {26a}

Trained researchers will inform the patients about the details of the trial and explain any potential adverse outcomes. Informed consent forms have been obtained from the parents of all infants participating in the trial.

### Additional consent provisions for collection and use of participant data and biological specimens {26b}

Regarding the collection and use of participant data, these data will be collected through standardized procedures. If any follow-up studies or additional analyses related to this research are conducted in the future, the data will be retrieved and used for those studies. Furthermore, access to these data will be restricted to authorized personnel and will only be used for the purposes outlined in the informed consent form. As for biological specimens, this study does not involve the collection of biological samples, as all data are obtained through non-invasive clinical monitoring procedures, in line with the standards for observational research.

## Interventions

### Intervention description {11a}

Randomization will be performed immediately upon confirmation of eligibility. Following group allocation, infants will commence PS therapy according to their assigned intervention protocol.

#### Artificial airway

LMA size 1, 1.5 (classic);

Endotracheal tube without cuff.

#### Pulmonary surfactant (PS)

All patients will receive the same PS preparation.

#### Premedication

In the LMA group, all patients underwent a standardized pre-procedural protocol involving the application of lidocaine spray for effective surface anesthesia. This preparatory step was implemented to ensure patient comfort and optimize the conditions for successful LMA placement and subsequent pulmonary surfactant administration. The use of topical lidocaine, a well-established local anesthetic agent, served to minimize potential discomfort and airway reflexes during the procedure, thereby enhancing the overall safety and tolerability of this noninvasive approach.

### Operating procedure

#### Experimental group: LMA group

1. Suction secretions and position the patient correctly.
2. Administer lidocaine spray for topical anesthesia.
3. Insert the LMA.
4. Confirm the correct position of the LMA by observing chest movement, auscultating the lungs, and monitoring SpO_2_ for 1 minute.
5. Administer PS through the LMA.
6. Use a T-piece to provide high-frequency, low tidal volume positive pressure ventilation concurrently.
7. During the procedure, confirm the uniform distribution of PS by auscultation.
8. Periodically aspirate the cuff to confirm the LMA seal.
9. After the PS administration, continue positive pressure ventilation until no significant wet crackles are heard upon auscultation.
10. Remove the LMA and continue noninvasive ventilation.

#### Control group: INSURE group

1. Suction secretions and position the patient correctly.
2. Perform endotracheal intubation, then connect to the T-piece for positive pressure ventilation.
3. Confirm the correct position of the tube by auscultation, observing chest movement, checking for condensation on the tube, and monitoring SpO_2_ for 1 minute.
4. Administer PS through the endotracheal tube.
5. Continuously use the bag-valve-mask for high-frequency, low tidal volume positive pressure ventilation.
6. During the procedure, confirm the uniform distribution of PS by auscultation.
7. After PS administration, continue positive pressure ventilation with the T-piece until no significant wet crackles are heard upon auscultation.
8. Remove the endotracheal tube and continue noninvasive ventilator support.

### Criteria for discontinuing or modifying allocated interventions {11b}

Intervention will be terminated if, during or after the procedure, the infant experiences unrelieved worsening of respiratory distress, severe hypoxia, or a significant increase in ventilator settings requiring endotracheal intubation or prolonged intubation. If the guardian of an enrolled infant requests to withdraw from the study, their decision will be fully respected.

### Strategies to improve adherence to interventions {11c}

Before the study begins, the research team will undergo protocol-based training to ensure a clear understanding of their respective roles and responsibilities. Pediatricians will assess whether the infant meets the criteria for PS administration, respiratory therapists will be responsible for the intervention and data recording, and nurses will assist with the intervention. Data recorders will also receive training to ensure consistency in data collection. Any challenges encountered during the trial will be promptly addressed.

### Relevant concomitant care permitted or prohibited during the trial {11d}

All enrolled infants will receive standardized clinical care in accordance with the established neonatal intensive care protocols throughout the study period, with all necessary therapeutic interventions fully maintained. During the intervention, standard supportive procedures will be followed as required.

### Ancillary and post-trial care {30}

Participants will receive necessary post-trial care based on their clinical needs, including follow-up for any trial-related adverse effects. Compensation for harm caused by trial participation will be provided according to ethical guidelines and local regulations, covering medical expenses and related costs. Participants will be informed of these provisions before enrollment, and ongoing medical support will be arranged if needed.

### Outcomes {12}

#### Primary outcome measures

The SF index (SpO_2_/FiO_2_) at 1 day after PS administration.

### Secondary outcome measures

1. The SF index before and after the administration of PS.
  The main monitoring time points are: before PS use and at 1 minute, 3 hours, 6 hours, and 12 hours after PS administration.
2. Time taken for placement.
3. Number of SpO_2_ fluctuations, episodes of bradycardia, and tachycardia during intubation.
4. Whether PS could be aspirated from the stomach after administration.
5. Administration of a second dose of PS due to NRDS (administered in the same manner as the first dose) (ethically assessed).
6. Incidence of pneumothorax.

### Participant timeline {13}

**Table:**
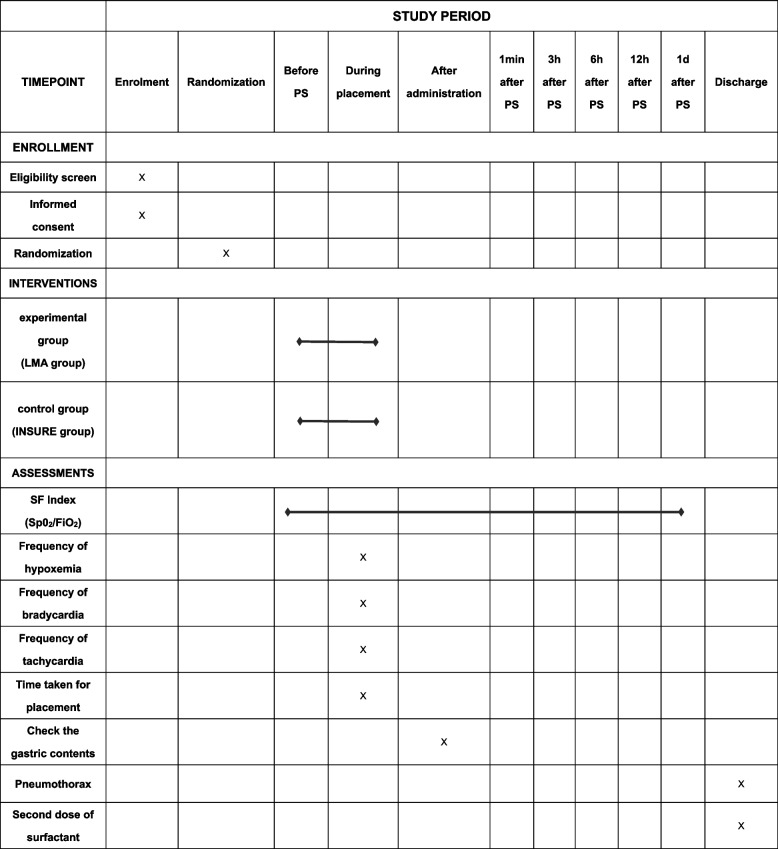

### Sample size {14}

The sample size for this equivalence trial was determined a priori based on the primary outcome—the SF index at 1 day after PS administration. The calculation was performed using EmpowerStats (www.empowerstats.com) and R software (version 4.1.0, R Foundation for Statistical Computing, Vienna, Austria). Based on pre-experimental data, the mean SF index was 4.5 in both the LMA and INSURE groups, with standard deviations of 0.5 and 0.3, respectively, yielding a pooled SD of approximately 0.41. For this equivalence design, the sample size was determined according to a predefined equivalence margin (Δ) of 0.45, which was considered clinically acceptable by expert consensus as it represents less than a 10% difference from the baseline mean value.

Using these parameters (*Δ* = 0.45, SD = 0.41, *α* = 0.05, power = 90%), a two one-sided test (TOST) procedure indicated that a minimum of 23 participants per group was required. Allowing for an anticipated 20% dropout rate, we planned to enroll 29 participants per group, for a total of 58 infants. To facilitate recruitment, the final target sample size was rounded to 60 infants (30 per group).

This methodological approach underscores our commitment to rigorous scientific standards and enhances the reliability and validity of our trial outcomes.

### Recruitment {15}

This study will be conducted at West China Second Hospital, a teaching hospital in Chengdu, China. After the admission of infants who may meet the eligibility criteria, the principal investigator or a delegated deputy will explain the written informed consent to at least one parent. A senior investigator will be available at all times to address any concerns raised by parents or clinicians during the course of the trial.

## Assignment of interventions: allocation

### Allocation—sequence generation {16a} {16b}

After meticulous screening to ensure compliance with the inclusion criteria, eligible infants will be systematically randomized into one of two distinct groups: the LMA group or the INSURE group, adhering to a 1:1 allocation ratio. The randomization sequence will be computer-generated using R software (version 4.1.0, R Foundation for Statistical Computing, Vienna, Austria). To maintain allocation concealment, the randomization will be performed using sealed envelopes. Blocking will be used to enhance the balance and integrity of group assignments. In scenarios involving multiple births, each infant will undergo individual randomization to preserve the independence and validity of the study outcomes. This comprehensive approach underscores our commitment to methodological precision and the reliability of our clinical trial.

### Implementation {16c}

Upon obtaining consent, trained research coordinators will sequentially open the numbered randomization envelopes to reveal group assignments. A protocol-certified respiratory therapist will then administer the designated treatment intervention following standardized procedures. During the enrollment process, the therapist will not be blinded to the group allocation.

## Assignment of interventions: blinding

### Who will be blinded {17a}

Healthcare staff: Blinding is not feasible.

Patients and their families: Blinded.

Nurses: Blinding is not feasible.

Outcome assessors: Blinded.

Biostatisticians: Blinded.

### Procedure for unblinding if needed {17b}

Blinding was implemented for both outcome assessors and biostatisticians in this trial. Outcome assessors remained blinded throughout the processes of data collection and outcome evaluation to ensure unbiased assessment. Biostatisticians were also blinded during data analysis to minimize analytical bias. In contrast, all clinical personnel and participants were aware of the treatment allocations to preserve the authenticity and integrity of the pragmatic intervention conditions.

## Data collection and management

### Data collection methods {18a} {18b} {19}

In this trial, we have implemented a structured approach to ensure the accuracy and reliability of data collection. A standardized bedside recording form will be utilized throughout the study period. During PS administration, 2–3 designated healthcare staff members will be responsible for conducting the procedure to maintain consistency, while an additional healthcare staff member will oversee the process and meticulously record the data. This dual-layered approach not only enhances the precision of data collection but also minimizes potential errors.

After the use of PS, the monitoring of primary and secondary outcomes will continue, with bedside nurses recording data at several predetermined time points: 1 minutes, 3 hours, 6 hours, 12 hours, and 1 day after PS administration. This ongoing monitoring ensures that any post-trial developments are captured systematically. Furthermore, to ensure the integrity and comprehensiveness of the dataset, the trial leader will retrospectively review and complete any additional data entries. This meticulous methodology underscores our commitment to rigorous data management and the reliability of the trial outcomes. By employing a combination of real-time monitoring and retrospective data completion, we aim to provide a robust and comprehensive dataset that accurately reflects the trial’s findings. This approach not only enhances the validity of our results but also ensures that all relevant data points are captured and analyzed thoroughly.

The trial data will undergo rigorous verification and management under the supervision of the principal investigator (PI). To ensure data integrity and security, all collected data will be stored in a designated area within the department, specifically allocated for this trial. Upon the completion of data collection, the PI will be responsible for summarizing and organizing the data into a structured format, facilitating subsequent analysis and reporting. In addition to data management, the PI will play a pivotal role in maintaining the study’s adherence to protocols. This includes conducting regular training sessions for all trial participants, focusing on procedural accuracy and standardized data collection methods. These training initiatives aim to minimize errors, enhance consistency, and ensure that all participants are well-equipped to follow the study’s guidelines meticulously.

By combining meticulous data management with ongoing participant training, this approach underscores the commitment to maintaining the highest standards of research quality and protocol compliance throughout the trial. This structured methodology not only safeguards the reliability of the data but also reinforces the overall credibility and validity of the study’s findings.

### Confidentiality {27}

The Data Safety and Monitoring Committee, consisting of two principal investigators, is responsible for storing and verifying the authenticity and integrity of the data to ensure the accuracy of the study results.

All participants will be identified by a reference number, which will be used throughout the study. Data will be securely protected during transmission and archiving to ensure patient privacy and prevent data leakage or loss. Any information containing names or other personal identifiers (e.g. consent forms and demographic tables) will be stored separately from the study records identified by the reference number. Contact information will be retained after obtaining consent from the family, for potential follow-up studies (if applicable).

### Biological sample item {33}

Not applicable to this test.

## Statistical methods

### Statistical analysis {20a}

All analyses will be performed using SPSS software (IBM Corp., Armonk, NY, USA). Continuous variables will be summarized as means ± SD or medians (IQR), and categorical variables as *n* (%). The primary outcome, the SF index at 1 day after PS administration, will be evaluated for clinical equivalence between the LMA and INSURE groups using the TOST procedure based on an independent-samples t-test. Equivalence will be concluded if the entire 90% CI of the mean difference lies within the predefined margin of − 0.45 to + 0.45; if normality is not met, a nonparametric test will be applied. Secondary outcomes will be analyzed using t-tests or Mann–Whitney U tests for continuous variables and logistic regression or Fisher’s exact test for binary variables, as appropriate. If baseline imbalances are identified, multivariable linear or logistic regression will be performed to obtain adjusted estimates. A two-sided *P*-value < 0.05 will be considered statistically significant.

### Interim analyses {21b}

In alignment with the predefined statistical analysis plan, this trial does not include provisions for interim analyses of the outcomes. By adhering to this approach, the trial prioritizes the accuracy, reliability, and ethical integrity of its findings, ultimately contributing to the generation of high-quality evidence to inform clinical practice.

### Statistical methods—additional analyses {20b}

This trial did not involve other analyses such as subgroup analyses.

### Statistical methods—analysis population and missing data {20c}

The primary analysis will follow the intention-to-treat (ITT) principle. The ITT population will include all randomized participants, regardless of whether they received the allocated intervention, deviated from the study protocol, or withdrew from the study. Participants with missing data for the primary outcome will be handled using multiple imputation methods. A per-protocol (PP) analysis, which will include only participants who completed the study without major protocol violations, will be performed as a sensitivity analysis.

### Plans to give access to the full protocol, participant level-data and statistical code {31c}

Following the publication of primary endpoints, de-identified research datasets will be made available to qualified investigators upon formal request to the corresponding author, in compliance with institutional data-sharing agreements and applicable privacy regulations.

## Oversight and monitoring

### Composition of the coordinating center and trial steering committee {5d}

The coordinating center consists of a principal investigator, a data manager, and other research staff. The principal investigator oversees the experiment, providing overall direction, while the data manager is responsible for recording, organizing, and reviewing the data. Other research staff actively participate in the trial. Regular meetings will be held throughout the study to monitor progress, provide feedback on trial conditions, and convene emergency meetings if necessary. This study does not include a trial steering committee or stakeholder/public involvement group.

### Data monitoring—formal committee {21a}

The research team will conduct weekly progress meetings throughout the study duration. The Executive Committee (EC), comprising principal investigators (HW, AYW, CC, YH, WLL, KT, RZ, ZY, JZ) working in conjunction with the study statistician (HW), will coordinate key operational areas.The EC will oversee various aspects of the study, including the implementation of all policies and the day-to-day operations.

### Adverse events/side effects {22}

Throughout the trial, the safety of all enrolled infants will be closely monitored. Both expected and unexpected adverse events (AEs) will be systematically collected and coded using MedDRA. All AEs will be actively identified through routine clinical assessments and medical record reviews, and classified by the investigator according to severity, duration, and relationship to the study intervention. Serious adverse events (SAEs), including any suspected unexpected serious adverse reactions (SUSARs), will be documented and reported to the institutional ethics committee within 24 hours of awareness, following standard operating procedures. For publication consistency, only the incidence of predefined expected AEs (e.g., pneumothorax) will be reported.

### Frequency and plans for auditing trial conduct {23}

Audits will be conducted periodically throughout the study to ensure compliance with the protocol, regulatory requirements, and ethical standards. The frequency of audits will be determined based on study milestones. Independent auditors will oversee the trial’s conduct, focusing on data integrity, participant safety, and protocol adherence. Audit findings will be reported to the principal investigator as needed.

### Protocol amendments {25}

Any modifications to the protocol during the study, including changes to study design, eligibility criteria, outcomes, analyses, patient population, sample size, or study procedures, will require a formal amendment. This amendment must be submitted to the ethics committee for review and approval.

### Dissemination {31a} {31c}

Trial results will be communicated to participants, healthcare professionals, and the public through publication in peer-reviewed journals, presentations at scientific conferences, and reporting in clinical trial registries. Participants will be informed of the outcomes at study completion. Data will be anonymized to protect confidentiality, and there will be no restrictions on publication. The principal investigator will ensure transparent and timely dissemination of results.

## Discussion

The OI (oxygenation index [FiO_2_ × Paw/PaO_2_]) and the P/F (PaO_2_/FiO_2_ ratio) are crucial for assessing the severity of NRDS and determining oxygenation levels. However, the PaO_2_ requires arterial blood gas sampling, which presents a challenge for infants who are smaller and more fragile. Several studies have shown a correlation between the SF index and P/F, and the SF index does not require invasive blood sampling [16, 17]. It can be rapidly obtained at any clinical node and allows for continuous monitoring of neonatal oxygenation, making it a promising tool with broader clinical application potential.

In this trial, we introduced the innovative use of the SF index to assess oxygenation levels in infants before and after PS administration using two methods. This enabled real-time evaluation of PS administration effectiveness. We also monitored key factors such as artificial airway placement time and episodes of hypoxemia, bradycardia, and tachycardia. Additionally, we compared the operation duration between the two methods and tracked adverse events to evaluate the safety and outcomes of both approaches in treating NRDS.

Since the laryngeal mask is positioned above the vocal cords, there may be a higher risk of drug wastage during its use. Therefore, we also measured the amount of PS residue in the stomach after use. Furthermore, we monitored the incidence of repeated PS use and pneumothorax to compare the effects and prognosis of the two methods in treating NRDS.

This trial, which compares the effectiveness of the LMA and INSURE method for administering PS, aims to gather additional data supporting the non-invasive use of PS and validate its clinical efficacy.

## Trial status

Protocol version: Version 2, Aug 11, 2023.

Recruitment start date: Oct 20, 2023.

Recruitment completion date: Jun 1, 2026 (estimated).

## Data Availability

For the final study, datasets used and/or analyzed during the study period will be made available upon reasonable request to the coordinating researchers. The data provided will be anonymized participant data, accompanied by a data dictionary, and will be limited to the data presented in the manuscript.

## Abbreviations

NRDS: Neonatal respiratory distress syndrome
RDS: Respiratory distress syndrome
PS: Pulmonary surfactant
INSURE: Intubation-surfactant-extubation
LMA: Laryngeal mask airway
nCPAP: Nasal continuous positive airway pressure
NIPPV: Non-invasive positive pressure ventilation
HFNC: High-flow nasal cannula
FiO_2_: Fraction of inspired oxygen
SpO_2_: Pulse oximetry oxygen saturation
TOST: Two one-sided test
CI: Confidence interval
PI: Principal investigator
ITT: Intention-to-treat
PP: Per-protocol
AEs: Adverse events
SAEs: Serious adverse events
SUSARs: Serious adverse reactions
OI: Oxygenation index [FiO_2_ × Paw/PaO_2_]
PaO_2_: Partial pressure of oxygen in arterial blood
Paw: Peak airway pressure
P/F: The arterial partial pressure of oxygen (PaO_2_) to fraction of inspired oxygen ratio (FiO_2_)
ICMJE: International Committee of Medical Journal Editors
